# A recombinant protein vaccine induces protective immunity against SARS-CoV-2 JN.1 and XBB-lineage subvariants

**DOI:** 10.1038/s41392-025-02154-6

**Published:** 2025-02-26

**Authors:** Jingyun Yang, Weiqi Hong, Huashan Shi, Zhenling Wang, Cai He, Hong Lei, Hong Yan, Aqu Alu, Danyi Ao, Zimin Chen, Yanan Zhou, Hao Yang, Yun Yang, Wenhai Yu, Cong Tang, Junbin Wang, Bai Li, Qing Huang, Hongbo Hu, Wei Cheng, Haohao Dong, Jian Lei, Lu Chen, Xikun Zhou, Li Yang, Wei Wang, Guobo Shen, Jinliang Yang, Zhiwei Zhao, Xiangrong Song, Qiangming Sun, Youchun Wang, Shuaiyao Lu, Jiong Li, Guangwen Lu, Weimin Li, Yuquan Wei, Xiawei Wei

**Affiliations:** 1 Laboratory of Aging Research and Cancer Drug Target, State Key Laboratory of Biotherapy and Cancer Center, National Clinical Research Center for Geriatrics, West China Hospital, Sichuan University, Chengdu, Sichuan China; 2 National Kunming High-level Biosafety Primate Research Center, Institute of Medical Biology, Chinese Academy of Medical Sciences and Peking Union Medical College, Yunnan, China; 3 Department of Respiratory and Critical Care Medicine, Med-X Center for Manufacturing, Center of Precision Medicine, Precision Medicine Key Laboratory of Sichuan Province, Frontiers Science Center for Disease-related Molecular Network, West China Hospital, West China Medical School, Sichuan University, Chengdu, China

**Keywords:** Vaccines, Infectious diseases

## Abstract

The emergence of XBB- and JN.1-lineages with remarkable immune evasion characteristics have led to rises in breakthrough infections within populations. In addition, the unfavorable impacts of immune imprinting, stemming from continuous exposure to antigens from circulated viruses, have been observed to incline immune response against earlier lineages, thereby declining the neutralization to newly emerged Omicron subvariants. In response to this, the advancement of next-generation vaccines against COVID-19 targeting components from new subvariants such as XBB-lineage is imperative. In the current study, a self-assembled trimeric recombinant protein (RBD_XBB.1.5_-HR) was generated by concatenating the sequences of the receptor binding domain (RBD) derived from XBB.1.5 with heptad-repeat 1 (HR1) and HR2 sequences from the spike S2 subunit. Adjuvanted-RBD_XBB.1.5_-HR induced robust humoral and cellular immune responses, characterized by elevated neutralization against JN.1-inculuded subvariants and a substantial population of antigen-specific T memory cells. Protective immunity conferred by RBD_XBB.1.5_-HR vaccine was preserved post-immunization, as evidenced by germinal center B (GC B) and T follicular helper (Tfh) responses, sustained neutralization potency, and an increase in memory B cells (MBCs) and long-lived plasma cells (LLPCs). The RBD_XBB.1.5_-HR vaccine showed a favorable boosting effect when administered heterologously after three doses of inactivated virus (IV) and mRNA vaccines. Significantly, it provided protection against live Omicron EG.5.1 viruses in vivo. The monovalent RBD_XBB.1.5_-HR vaccine showed favorable safety and immunogenicity, boosting neutralizing antibodies against JN.1- and XBB-lineage subvariants in individuals with prior COVID-19 vaccinations. These findings highlight its clinical potential in safeguarding against circulating Omicron subvariants.

## Introduction

Since first identified in late 2021, the Omicron BA.1 variant of SARS-CoV-2 has marked a profound shift in the trajectory of viral evolution. Rapidly becoming the dominant strain, Omicron spread globally, giving rise to a multitude of descendant subvariants, which fueled widespread infections. By early 2023, the springing of the XBB lineages, marked by enhanced immune evasion, further propelled the evolution of various subvariants.^1^ Notable among these are XBB.1.5 and XBB.1.16, which have rapidly disseminated across regions. In the wave of XBB.1.5, two of its descendant subvariants, EG.5 and EG.5.1, have experienced remarkable global expansion. Other XBB-derived lineages, including HK.3, FL.1.5.1, and HV.1have also surfaced,^2–5^ engaging in a competitive dance for dominance in different regions. Alongside the XBB lineages, a new subvariant, namely BA.2.86, was detected for the first time in August 2023.^6,7^ Distinguished by significant mutation sites in the spike protein compared to both earlier BA.2 and later XBB.1.5, BA.2.86 raised serious concerns regarding its potential for immune escape. More recently, a descendant of BA.2.86, named JN.1,^8–10^ carrying L455S mutationsite in the receptor-binding domain (RBD), has emerged as the dominant circulating variant globally. Its more recent descendants, KP.3.1.1 and XEC are the latest leading variants, continuing the rapid global spread.

Though the World Health Organization (WHO) declared to end the emergency phase of COVID-19 pandemic, the persistent threat of virus reinfections underscores the critical importance of widespread vaccination in mitigating the risks associated with symptomatic infection, severe disease, and even mortality. Nevertheless, the complex hybrid immune background in individuals, shaped by diverse factors such as vaccination history, infection experiences, the presence of a variety of infectious variants, and the temporal gaps between vaccination and infection, brings ever-increasing challenges to the development of next-generation boosters.^11,12^ On one hand, the newly emerged subvariants have demonstrated pronounced abilities for immune evasion, substantially impairing neutralization in individuals who have received booster or suffered breakthrough infections with pre-XBB-lineage subvariants.^1,13–16^ On the other hand, the phenomenon of immune imprinting must be considered during vaccine development, such repeated prior antigen exposure can hinder the magnitude of neutralizing antibody response to new Omicron subvariants.^17,18^ Experimental studies in animal models have demonstrated vaccination-induced imprinting immunity, highlighting its relevance in the development of human vaccines.^12^ In addition, studies indicate that repeated vaccinations with mRNA vaccines or inactivated vaccines containing the Spike antigen from earlier variants tend to induce immune responses against those earlier lineage variants while decreasing immune responses toward more recent circulating Omicron subvariants.^19,20^ Similarly, BA.4-BA.5 bivalent vaccines only augment the immune response toward Omicron subvariants in a limited extent.^21^ These findings underscore the pressing need to reassess the composition of current vaccines and to expedite the advancement of COVID-19 vaccines specifically targeting the evolving XBB lineages.

In June 2023, the U.S. Food and Drug Administration (FDA) recommended the use of monovalent XBB descendant as primary antigen of COVID-19 vaccines, leading to the approval of monovalent XBB.1.5 based mRNA vaccines by developers including Moderna and Pfizer/BioNTech,^18^ which have shown robust neutralization against the subvariants from XBB- and JN.1-lineage.^22–26^ Our research has previously demonstrated that vaccination with an XBB.1.5 variant derived antigen provides cross-neutralization against various Omicron variants when compared to those from other Omicron strains.^27^ Despite the availability of these XBB-derived mRNA vaccines, some previous studies suggested that recombinant subunit protein vaccines might offer significant advantages as heterologous boosters following immunizations with inactivated vaccine (IV) or mRNA vaccines.^28–30^ Responding to this potential, both our team and Novavax^25,31^ have developed adjuvanted recombinant protein vaccines utilizing an updated XBB.1.5 antigen. Notably, our XBB.1.5 recombinant protein monovalent vaccine has already been granted emergency use authorization in mainland China.

In the current study, we describe the design of our trimeric antigen and report the results of the immunogenicity assay for our XBB.1.5 monovalent protein vaccine from preclinical animal studies, as well as safety and immunogenicity evaluation in human subjects from an investigator-initiated clinical trial (IIT). We harnessed the self-assembly characteristics of spike heptad-repeat sequences^32^ to generate an XBB.1.5 RBD-derived recombinant trimeric protein (RBD_XBB.1.5_-HR) on an insect cell (Sf9) protein expression platform. We incorporated an MF59-like oil-in-water adjuvant to formulate the XBB.1.5 monovalent protein vaccine. Intramuscular immunization with the adjuvanted RBD_XBB.1.5_-HR recombinant vaccine provoked robust and enduring humoral and cellular immune responses against JN.1 and XBB-lineage variants. Heterologous vaccination with the protein-based RBD_XBB.1.5_-HR vaccine, following three doses of inactive virus or mRNA vaccines, improved neutralizing activities in animal models. The monovalent RBD_XBB.1.5_-HR vaccine bestowed efficient protective immunity against the challenge of live Omicron EG.5.1 viruses in mice. Notably, the monovalent RBD_XBB.1.5_-HR COVID-19 vaccine showed good tolerability and safety in human participants, and induced sera cross-neutralizing antibodies against all XBB- and JN.1- lineage subvariants. These results suggest that the monovalent RBD_XBB.1.5_-HR protein vaccine represents a favorable candidate of COVID-19 booster for clinical use.

## Results

### RBD_XBB.1.5_-HR vaccine triggered robust humoral response in mice and rats

In our previous study, we reported a self-assembled trimeric protein developed based on the ability of peptides containing the spike HR1 and HR2 regions to spontaneously form a 6-helix bundle (6-HD) higher-order structure.^32^ In the current study, we applied the same design strategy, directly linking the XBB.1.5-derived RBD (amino acids V320-G545) with the HR1 (L916-L966) and HR2 (K1157-L1203) sequences from the spike S2 subunit in a tandem arrangement. The resulting recombinant trimeric protein, named RBD_XBB.1.5_-HR, was subsequently expressed using the Bac-to-Bac Baculovirus Expression System and finally purified to a high degree of homogeneity, as described in previous reports.^32,33^

**Fig. 1:**
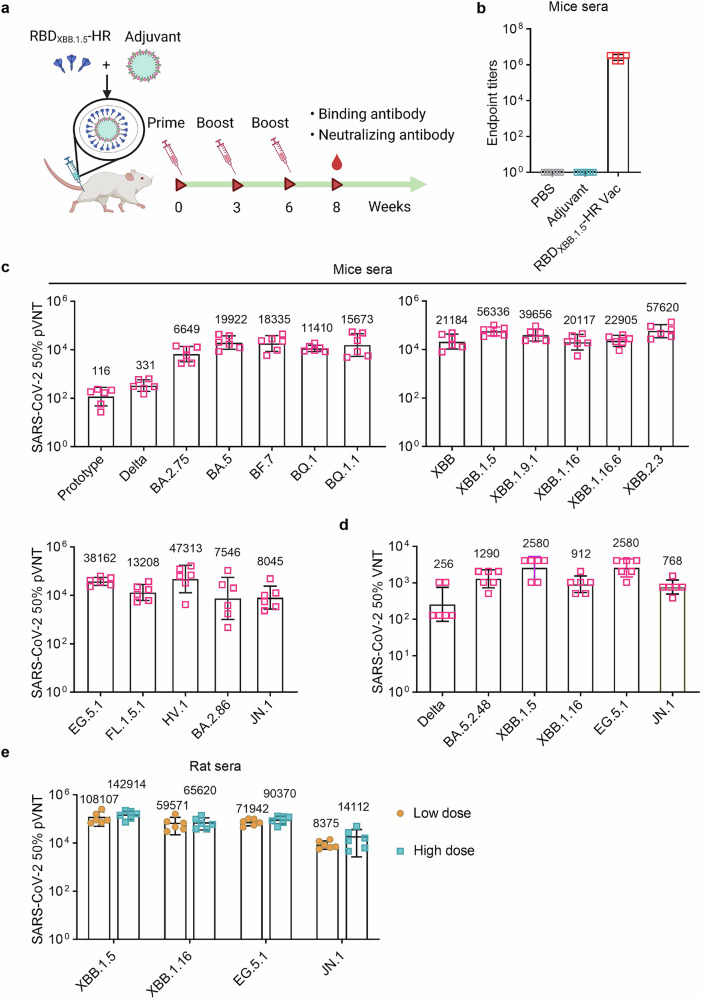
RBD_XBB.1.5_-HR vaccine induces strong humoral immune response in mice and rats. **a** The schematic visualization of immunization schedule. NIH mice were intramuscularly immunized three times with PBS, adjuvant and 10 μg of RBD_XBB.1.5_-HR proteins formulated with MF59-like adjuvant at 0, 3, and 6 weeks, the sera samples were collected at 8 weeks. **b** Sera RBD_XBB.1.5_-HR antigen-specific binding antibody titers in mice (*n* = 6 mice per group). **c**, **d** Neutralizing antibodies against a variety of pseudoviruses (**c**) and several authentic viruses (**d**) in mice sera samples (*n* = 6 mice per group). **e** Neutralizing antibodies against XBB.1.5, XBB.1.16, EG.5.1 and JN.1 pseudoviruses by sera samples from vaccinated SD rats immunized with 30 μg and 60 μg RBD_XBB.1.5_-HR vaccine (*n* = 6 rats per group). pVNT, pseudovirus neutralizing antibody titer, VNT, virus neutralizing antibody titer. Data are shown as geometric mean values accompanied by SD in (**b**–**e**)

To determine the immunogenicity of RBD_XBB.1.5_-HR vaccine, National Institute of Health-Swiss (NIH) mice were vaccinated intramuscularly with of 10 μg oil-in-water MF59 adjuvanted-RBD_XBB.1.5_-HR proteins following a 21-day spaced prime-boost regimen for 3 times. Samples were collected at 8 weeks to evaluate the immune response induced by vaccination (Fig. 1a). High titers of RBD_XBB.1.5_-HR protein-specific antibodies were observed in serum samples from vaccinated mice (Fig. 1b). Neutralizing activity conferred by the monovalent RBD_XBB.1.5_-HR vaccine was subsequently assessed through pseudovirus neutralization assays (Fig. 1c). The vaccine demonstrated high potency of neutralization against all XBB-lineage subvariants. 50% neutralization geometric mean titers (GMTs) against pseudoviruses of XBB, XBB.1.5, XBB.1.9.1, XBB.1.16, XBB.1.16.6, and XBB.2.3 variants were determined as 21184, 56336, 39656, 20117, 22905, and 57620, respectively. The vaccine also exhibited efficient neutralization against EG.5.1, FL.1.5.1, and HV.1 pseudoviruses, representing several XBB-lineage descendants circulated globally. In addition, RBD_XBB.1.5_-HR recombinant vaccine induced mutual neutralization against other Omicron variants, including BA.2.75, BA.5, BQ.1, BF.7, and BQ.1.1, aligning with our prior findings that antigens from XBB lineages induce neutralizing responses against multiple variants from Omicron sublineages. Of particular note, the emerged subvariants BA.2.86 and its descendant JN.1, characterized by significant mutations in spike protein sequences compared with previous Omicron BA.2 variant and XBB.1.5, raised concerns about their potential immune escape ability.^6,7,10^ Here, we found the 50% neutralization GMT against BA.2.86 and JN.1 subvariants were 7546 and 8045, respectively.

An authentic virus neutralization assay was further conducted to detect the neutralization levels in serum against several dominant circulating SARS-CoV-2 variants. The monovalent RBD_XBB.1.5_-HR vaccine elicited robust neutralizing responses, particularly against XBB.1.5 and EG.5.1 including XBB-lineage variants (Fig. 1d). GMTs against live XBB.1.5, XBB.1.16, and EG.5.1 were 2580, 912, and 2580, while GMTs against pre-XBB variants, such as Delta and BA.5.2.48, were 256 and 1290, respectively. The neutralization activity against the circulating JN.1 live virus was also assessed, with a GMT of 768.

The antibody response to the RBD_XBB.1.5_-HR vaccine in rats was also evaluated (Fig. 1e). Sprague-Dawley (SD) rats were intramuscularly vaccinated with 30 μg and 60 μg of RBD_XBB.1.5_-HR vaccine three times in the low-dose group and high-dose group, respectively, following a 21-day spaced prime-boost regimen. Serum samples from rats were collected at 8 weeks to assess the humoral immune responses induced by 3 vaccinations. The RBD_XBB.1.5_-HR vaccine elicited potent neutralizing responses against all XBB-lineage subvariants. 50% neutralization GMTs against XBB.1.5, XBB.1.16, and EG.5.1 were 108107, 59571, and 71942 in samples from rats receiving low-dose vaccine, and detected to be 142914, 65620, and 90370 in the high-dose group, respectively. GMTs for 50% neutralizing antibody titers against the JN.1 virus exceeded 8,000 in both groups, highlighting the vaccine’s efficient protection against this subvariant.

**Fig. 2:**
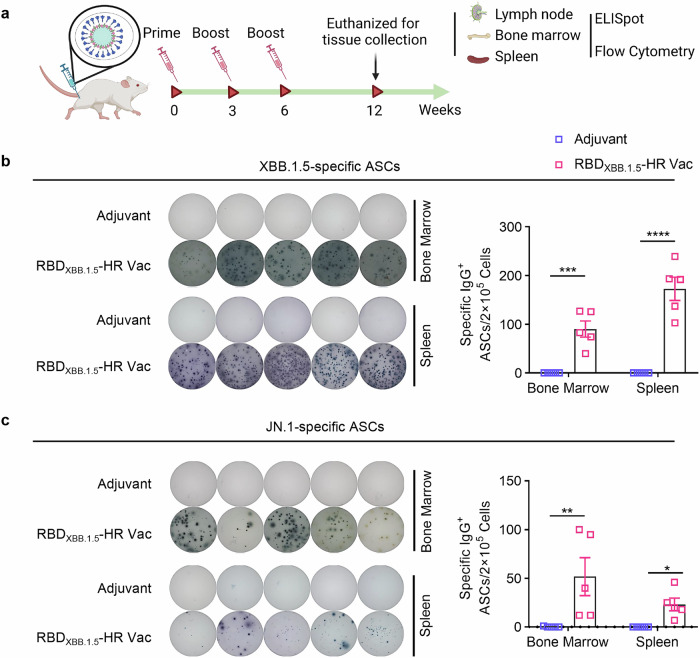
The RBD_XBB.1.5_-HR vaccine induces a large number of strong RBD-specific antibody-secreting cells (ASCs). **a** The schematic representation of immunization and sampling schedule. NIH mice were immunized with 10 μg of RBD_XBB.1.5_-HR vaccine three times intramuscularly at 0, 3, and 6 weeks, the mice were sacrificed and the tissue samples were collected at 12 weeks for assays. Visual representations and quantitative assessments of RBD-specific IgG antibody secreting cells (ASCs) against XBB.1.5 (**b**) and JN.1 (**c**) variants in bone marrow and spleen tissues (*n* = 5 mice per group). *P* values in (**b**, **c**) were carried out by Two-way ANOVA followed by Sidak’s multiple comparisons test. ****P < 0.0001; ***P < 0.001; **P < 0.01; *P < 0.05

We next assessed the frequencies of antibody-secreting cells (ASCs) in both bone marrow and spleen tissues at 6 weeks post-vaccination (Fig. 2a). Consistent with results from the neutralization assays, a significantly increased number of RBD_XBB.1.5_-specific IgG ASCs were observed in mice that received the adjuvanted RBD_XBB.1.5_-HR vaccine (Fig. 2b). Notably, ASCs responding to the JN.1 antigen (RBD_JN.1_) were also present post-vaccination, suggesting a cross-neutralizing response against this variant (Fig. 2c).

### RBD_XBB.1.5_-HR vaccine elicited strong cellular and germinal center responses

**Fig. 3:**
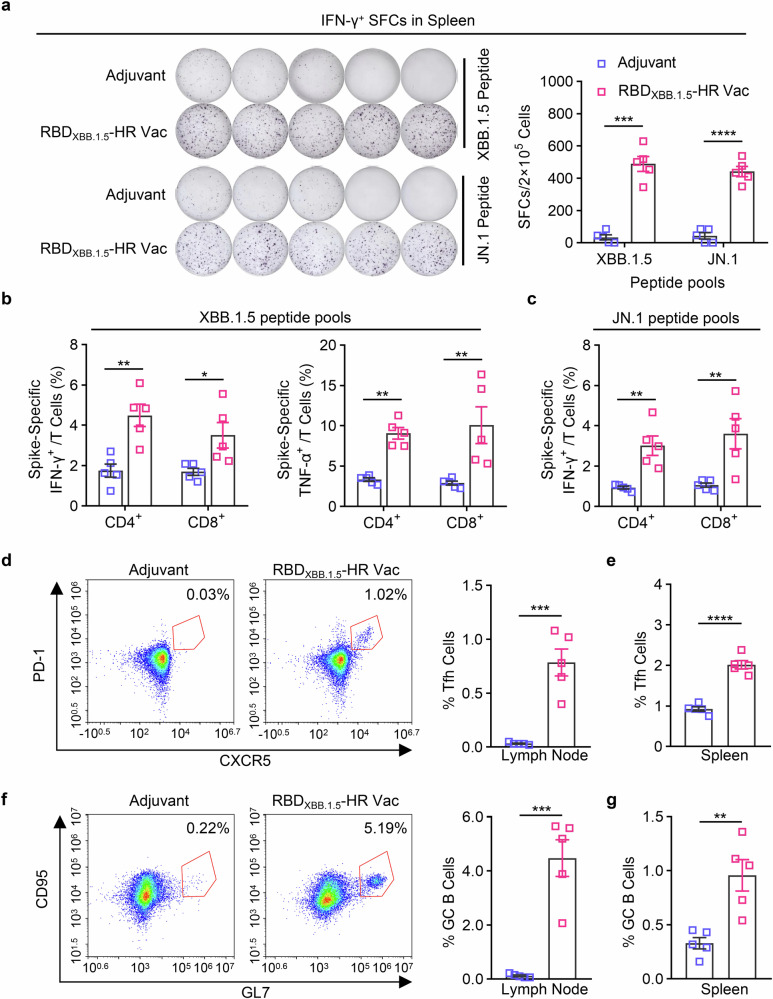
RBD_XBB.1.5_-HR vaccine induces strong cellular immune response in mice. **a** The representative images and quantitative analysis of IFN-γ-secreting lymphocytes in spleen after stimulation with peptides pools of XBB.1.5 and JN.1 full-length spike proteins (*n* = 5 mice per group). **b**, **c** the percentages of splenic IFN-γ- and TNF-α-secreting CD4^+^/CD8^+^ and CD44^+^ memory T cells after being stimulated by spike peptide pools covering full-length spike proteins of XBB.1.5 (**b**) and JN.1 (**c**) variants (*n* = 5 mice per group). The representative images and percentages of Tfh and GC B cells in inguinal lymph nodes (**d**, **f**) and spleen (**e**, **g**) tissues (*n* = 5 mice per group). Data are presented as mean with SEM. SFC, spot-forming cells; GC B, germinal center B cells; Tfh, T follicular helper cells. *P* values in (**a**–**c**) were conducted by Two-way ANOVA followed by Sidak’s multiple comparisons test, and in (**d**–**g**) were performed by Unpaired Student’s t-tests. ****P < 0.0001; ***P < 0.001; **P < 0.01; *P < 0.05

We next sought to evaluate the T and B cell response induced by our monovalent RBD_XBB.1.5_-HR vaccine. Lymphocytes from immunized spleen tissue were cocultured with peptide pools covering full-length spike protein of XBB.1.5 and JN.1 variants to detect the antigen-specific CD4^+^ and CD8^+^ T cell responses by enzyme linked immunospot (ELISpot) (Fig. 3a) and intracellular cytokines staining (ICS) (Fig. 3b, c) assays. Immunization with the RBD_XBB.1.5_-HR vaccine elicited a robust cellular immune response against antigens from the XBB.1.5 and JN.1 variants, as evidenced by notable increases in total number of IFN-γ-secreting cells (Fig. 3a) and higher percentages of IFN-γ and TNF-α-secreting, antigen-experienced memory CD8^+^ cells and CD4^+^ T cells (Fig. 3b, c) in the RBD_XBB.1.5_-HR vaccine group.

The T follicular helper (Tfh) cell and germinal center B (GC B) responses, critical for long-term protection against virus infection, were also assessed using flow cytometry.^34,35^ We observed elevated frequencies of Tfh (CD4^+^CXCR5^+^PD-1^+^) cells and GC B (CD19^+^GL7^+^CD95^+^) cells in inguinal lymph nodes (Fig. 3d, f) and spleen tissues (Fig. 3e, g), following immunization with the RBD_XBB.1.5_-HR vaccine, suggesting that our monovalent vaccine may confer long-term protective immunity.

### Long-term protective immunity induced by RBD_XBB.1.5_-HR vaccine

**Fig. 4:**
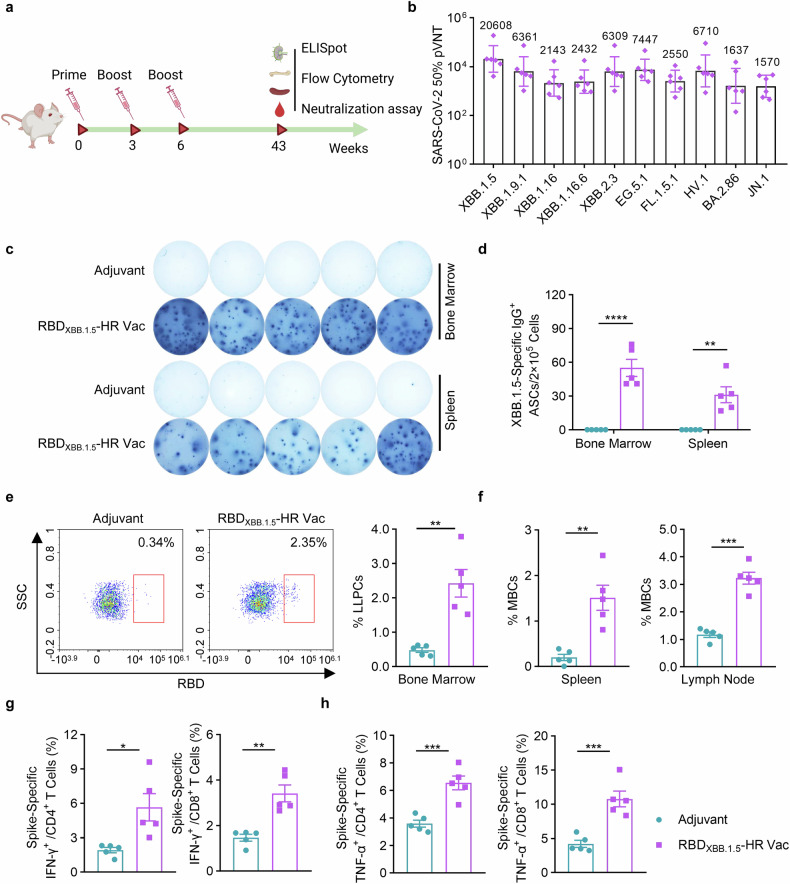
RBD_XBB.1.5_-HR vaccine induces long-term protective immune response. **a** The schematic representation of immunization schedule. NIH mice were intramuscularly injected with 10 μg of RBD_XBB.1.5_-HR proteins three times at 0, 3, and 6 weeks, the mice were sacrificed and the spleen, lymph node and bone marrow samples were collected at 43 weeks after the first immunization. **b** Neutralizing antibodies against a variety of pseudoviruses in serum samples from vaccinated mice (*n* = 6 mice per group). The Visual representations (**c**) and quantitative analysis (**d**) of RBD-specific IgG antibody secreting cells (ASCs) in bone marrow and spleen tissues (*n* = 5 mice per group). **e** The representative images and percentage of LLPCs in the bone marrow (*n* = 5 mice per group). **f** The percentage of MBCs in spleen and lymph nodes (*n* = 5 mice per group). The percentages of splenic IFN-γ- (**g**) and TNF-α- (**h**) secreting CD4^+^/CD8^+^ and CD44^+^ memory T cells after stimulation with peptide pools covered full-length spike proteins of XBB.1.5 variant (*n* = 5 mice per group). Data are presented as geometric mean values with SD in (**b**), and presented as mean with SEM in (**d**–**h**). LLPCs, long-lived plasma cells; *P* values in (**d**) were performed by the Two-way ANOVA followed by Sidak’s multiple comparisons test, and in (**e**–**h**) were conducted by Unpaired Student’s t-tests. ns, not significant. ****P < 0.0001; ***P < 0.001; **P < 0.01; *P < 0.05

A long-lasting antibody response is paramount for the establishment of protective immunity against COVID-19 disease. Consequently, our next objective was to evaluate the whether the neutralizing antibodies induced by the recombinant RBD_XBB.1.5_-HR vaccine could be retained over a 40-week period (Fig. 4a). The results of neutralization against pseudoviruses revealed that our adjuvanted RBD_XBB.1.5_-HR vaccine elicited prolonged neutralization lasting up to 43 weeks, with GMTs for XBB-lineage subvariants exceeding 2000, and over 1500 against BA.2.86 and JN.1 (Fig. 4b). Furthermore, antigen-specific ASCs were detectable in both bone marrows and spleens for an extended duration post-immunization (Fig. 4c, d).

The longevity of antibody production is deeply involved with the induction of somatically mutated memory B cells (MBCs) and long-lived plasma cells (LLPCs).^36,37^ Therefore, we assessed the percentages of these cells following vaccination (Fig. 4e, f). The RBD_XBB.1.5_-HR vaccine markedly induced LLPCs in bone marrow (Fig. 4e) and MBCs in spleen tissue and lymph nodes (Fig. 4f), which aligns with the notable induction of long-lasting sera neutralizing antibodies. Analogous to humoral immune response, persistence of cellular immune response was conferred by RBD_XBB.1.5_-HR vaccine, demonstrated by the presence of antigen-specific CD4^+^ and CD8^+^ T cells in spleen at 43 weeks post the initial immunization (Fig. 4g, h). These results demonstrate our monovalent RBD_XBB.1.5_-HR vaccine can afford long-term protection against SARS-CoV-2 XBB.1.5 variant.

### RBD_XBB.1.5_-HR vaccine can serve as a booster following mRNA and inactivated vaccine for heterologous immunization

Given the significant immune evasion displayed by XBB-lineage subvariants against immunity induced by prior vaccination and breakthrough infection, coupled with the unfavorable effect of imprinted immunity,^12,17–20^ the FDA has recommended to use monovalent XBB-lineage as the vaccine composition. Currently, both Moderna and Pfizer/BioNTech have received marketing authorization for the monovalent mRNA vaccine based on XBB.1.5 spike.^18^ However, some studies suggest that heterologous immunization with a recombinant protein-based booster may induce superior immune responses compared to homologs boost with inactive virus- or mRNA-based vaccines.^28–30^ Furthermore, the predominant COVID-19 vaccines administered in Chinese mainland are inactivated vaccines.^38^ Thus, we sought to evaluate the potential of the RBD_XBB.1.5_-HR vaccine to serve as a favorable booster shot for mRNA and inactivated vaccines.

**Fig. 5:**
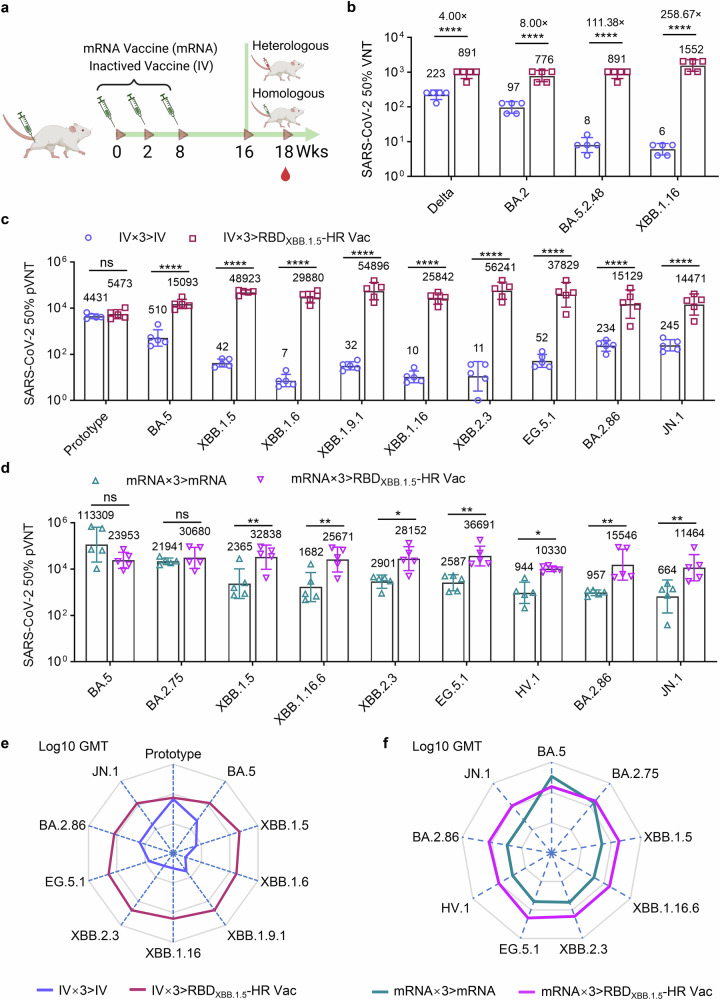
The RBD_XBB.1.5_-HR vaccine serves as a fourth booster, improving the neutralizing response following mRNA and inactivated vaccine immunization. **a**–**c** NIH mice were immunized three times with wildtype inactivate virus vaccine (IV), followed by another dose of homologous vaccination of inactivated vaccine (IV×3 > IV), or one heterologous injection of RBD_XBB.1.5_-HR vaccine (IV×3 > RBD_XBB.1.5_-HR Vac) (*n* = 5 mice per group). The neutralizing antibodies in serum samples against authentic viruses (**b**) and pseudotype variants of viruses (**c**) were determined. **d** NIH mice were immunized three doses of mRNA vaccine encoding full-length BA.5 spike, followed by one homologous booster vaccination of mRNA vaccine (mRNA×3>mRNA), or one heterologous injection of RBD_XBB.1.5_-HR vaccine (mRNA×3 > RBD_XBB.1.5_-HR Vac) (*n* = 5 mice per group), the sera neutralization against Omicron pseudoviruses were determined. **e**, **f** The radar charts were generated from the data of Log_10_ GMTs in (**c**, **d**) to reveal the tropism of neutralizing antibody response induced by homologous or heterologous vaccination. Data are presented as geometric mean values with SD in (**b**–**d**). *P* values in (**b**–**d**) were performed by the Two-way ANOVA followed by Sidak’s multiple comparisons test

We first immunized NIH mice three times with either inactivated vaccines based on the ancestral virus or an mRNA vaccines encoding full-spike protein based on the sequence of Omicron BA.5 variant, respectively. After 8 weeks, the mice received a homologous booster shot with either IV (IV×3 > IV group), mRNA vaccine (mRNA×3>mRNA group), or heterologous monovalent RBD_XBB.1.5_-HR vaccine (IV×3 > RBD_XBB.1.5_-HR Vac, and mRNA×3 > RBD_XBB.1.5_-HR Vac group) (Fig. 5a). In line with previous studies, XBB-included Omicron subvariants exhibited remarkable immune evasion to neutralization elicited by inactivated vaccine, and even the mRNA vaccine designed on the BA.5 sequence (Fig. 5b–d). Heterologous vaccination with RBD_XBB.1.5_-HR vaccine markedly restored neutralizing activities against all variants in the panels. We first performed the authentic virus neutralization assay to determine whether the RBD_XBB.1.5_-HR vaccine could represent a favorable fourth booster shot after three injections of inactivated virus vaccine (Fig. 5b). The GMTs in heterologous boosted mice serum samples against Delta, BA.2, BA.5.2.48 and XBB.1.16 live viruses reached 891, 776, 891, and 1552, respectively, demonstrating enhancements by 4.00-, 8.00-, 111.38-, and 258.67- fold, respectively, compared to mice receiving homologous booster.

Pseudovirus neutralization assay were then conducted to comprehensively assess the neutralizing responses induced by heterologous RBD_XBB.1.5_-HR vaccine vaccination as a booster shot (Fig. 5c, d). Omicron subvariants, particularly those within the XBB lineage, substantially compromised the neutralizing capacities elicited by both inactivated and mRNA vaccines. Remarkably, the group receiving RBD_XBB.1.5_-HR vaccine heterologous boost shot exhibited elevated levels of neutralizing antibodies against all XBB-lineage and BA.2.86-derived subvariants. In the heterologous vaccination group (IV×3 > RBD_XBB.1.5_-HR Vac) following inactivated vaccines, the GMTs against XBB.1.5, XBB.1.6, XBB.1.9.1, XBB.1.16, XBB.2.3, EG.5.1, BA.2.86 and JN.1 subvariants were determined to be 48923, 29880, 54896, 25842, 56241, 37829, 15129 and 14471, respectively. These values were enhanced by 1168.18-, 4139.36-, 1740.76-, 2472.39-, 5028.81-, 727.48-, 64.65- and 59.07-fold, respectively, in comparison to the IV group (IV×3 > IV) (Fig. 5c). Furthermore, in the group receiving three doses of mRNA vaccine followed by one dose of RBD_XBB.1.5_-HR vaccine (mRNA×3 > RBD_XBB.1.5_-HR Vac), the GMTs against XBB.1.5, XBB.1.16.6, XBB.2.3, EG.5.1, HV.1, BA.2.86, and JN.1 were determined to be 32838, 25671, 28152, 36691, 10330, 15546 and 11464, respectively, and the values were improved by 13.88-, 15.27-, 9.70-, 14.18-, 10.95-, 16.24- and 17.27-fold, respectively (Fig. 5d). Radar charts were generated from Log10 GMTs to clearly highlight the significant advantages of the neutralizing antibody response induced by heterologous vaccination with the RBD_XBB.1.5_-HR vaccine (Fig. 5e, f). These findings robustly affirm that the monovalent RBD_XBB.1.5_-HR vaccine represents a favorable candidate for a boosting shot, providing protection against variants inclusive of the JN.1, BA.2.86 and XBB lineages.

### RBD_XBB.1.5_-HR vaccine conferred effective protection against EG.5.1 virus challenge

**Fig. 6:**
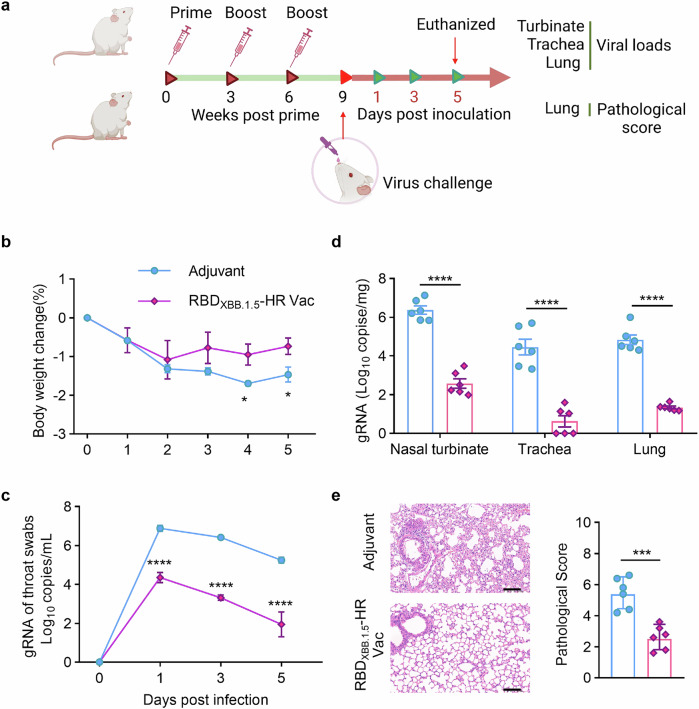
Monovalent RBD_XBB.1.5_-HR vaccine confers efficient protective immunity against the EG.5.1 infection in vivo. **a** The schematic representation of immunization and virus challenge schedule. NIH mice injected with adjuvant or 10 μg of adjuvanted RBD_XBB.1.5_-HR vaccine were intranasally challenged with live EG.5.1 viruses (1×10^6^ PFU/mouse, *n* = 6 mice per group). The fluctuations in body weight loss (**b**) and viral genomic RNA (gRNA) loads in throat swabs (**c**) were monitored daily. **d** The virus infected mice were all euthanized on day 5 post infection for tissue collection, the tissues of nasal turbinates, trachea and lung were collected to detect the loads of gRNA by RT-qPCR. **e** Histopathological changes and pathological score in the lung tissue from mice challenged with live EG.5.1 virus. Scale bars represent 100 μm in (**e**). Data are presented as mean with SEM in (**b**–**e**). *P* values in (**b** and **e**) were performed by Unpaired Student’s *t*-tests, in (**c**, **d**) were conducted by Two-way ANOVA followed by Sidak’s multiple comparisons test, ****P < 0.0001; ***P < 0.001; *P < 0.05

To assess protective efficacy of recombinant RBD_XBB.1.5_-HR vaccine in vivo, we challenged vaccinated mice with live EG.5.1 Omicron subvariant, a prevalent strain globally (20.2% of circulating strains in the US as of November 2023).^8^ Mice receiving 10 μg of RBD_XBB.1.5_-HR vaccine were challenged with 1×10^6^ plaque-forming units (PFU) of live Omicron EG.5.1 viruses intranasally, and daily monitoring ensued for bodyweight loss and alterations in viral genomic RNA loads in throat swabs (Fig. 6a). The control group exhibited some degree of weight loss on day 1 post-infection, reaching its maximum on day 4. In contrast, vaccinated mice experienced a brief weight reduction but quickly regained weight by day 2 (Fig. 6b). Additionally, viral RNA levels were markedly higher in the control group’s throat swabs, whereas the vaccinated mice exhibited a significant reduction in viral loads throughout the study (Fig. 6c).

On day 5 post-infection, tissues from upper and lower respiratory tract were collected for further analysis. In the control group, high loads of viral genomic RNA copies were detected in nasal turbinates, trachea, and lung tissues, while vaccinated mice showed a significant decrease in viral copies in these tissues (Fig. 6d). Pathological examination revealed mild lung damage in the control group, including consolidation, thickened alveolar septa, and small areas of inflammation (Fig. 6e). In contrast, lung tissues from the vaccinated mice appeared normal, with no signs of inflammation and intact alveolar structures (Fig. 6e). These findings demonstrate that the RBD_XBB.1.5_-HR vaccine is effective in triggering a robust immune response that protects against infection with the live EG.5.1 virus in respiratory tracts.

### RBD_XBB.1.5_-HR vaccine exhibited good safety and improved the neutralizing antibodies in human participants

To evaluate the clinical potential of the RBD_XBB.1.5_-HR vaccine, we enrolled participants and conducted an investigator-initiated clinical trial (IIT) to evaluate its tolerability and immunogenicity (Registration number: ChiCTR2300068248). The study received approval from the Biomedical Ethics Review Committee affiliated with West China Hospital, Sichuan University. and followed ethical guidelines.

In this study, participants were recruited with the requirement of meeting following inclusion criteria: (1) aged 18 years and above, with or without mild underlying diseases; (2) more than three months after immunization with a COVID-19 vaccine; (3) not infected or beyond 3 months since last infection. Participants were advised to monitor themselves, and those with an axillary temperature over 37.3 °C or an antigen positive test within the last 48 h were excluded. Each participant was aware of the research procedures and voluntarily signed informed consent. We finally recruited 54 human participants ranging in age from 21 to 71 into this clinical study, demographic characteristics of subjects were summarized in Table 1.

**Table 1:** Characteristics of participants

	Number of participants	Percentage
All participants	54	/
Age (range)		
Adult	35 (21-57)	64.8%
Elderly	19 (60-71)	35.2%
History of infection		
Uninfected	13	24.1%
Infected	41	75.9%
Health condition		
Healthy	39	72.2%
With underlying disease^a^	15	27.8%

^a^Underlying diseases include hypertension, hyperlipidemia, diabetes, bronchitis, myocardial infarction, gallstones, gallbladder polyps, gastrointestinal polyps, allergic rhinitis, seborrheic dermatitis

To evaluate the safety and tolerability of the monovalent RBD_XBB.1.5_-HR vaccine in humans was prescribed as primary objective in this clinical study. All participants received one dose (30 μg) of RBD_XBB.1.5_-HR vaccines, and the incidence of adverse reaction (AR) within 7 days after vaccination was monitored (Table 2). Within 7 days after injection, solicited adverse reactions were collected from a total of 7 participants (13.0%), with incidences of local and systemic adverse reactions being 11.2% and 1.9%, respectively. The local adverse reactions that occurred most frequently were pain (9.3%) and swelling (1.9%) at the injection site. Additionally, systemic reaction fever (1.9%) was reported by one case. Two participants reported unsolicited adverse reactions including acne (1.9%) and toothache (1.9%). All reported adverse reactions belonged to grades 1 and 2, and no adverse reactions of grade 3 or serious adverse events (SAEs) occurred in the trial.

**Table 2:** Adverse reaction of vaccine

		Number of participants	Percentage/%
Overall adverse reactions		9	16.7
Solicited adverse reactions within 7 days		7	13.0
Local	Pain at the injection site	5	9.3
	Swelling at the injection site	1	1.9
Systemic	Fever	1	1.9
Unsolicited adverse reactions within 7 days		2	3.7
	Acne	1	1.9
	Toothache	1	1.9

**Fig. 7:**
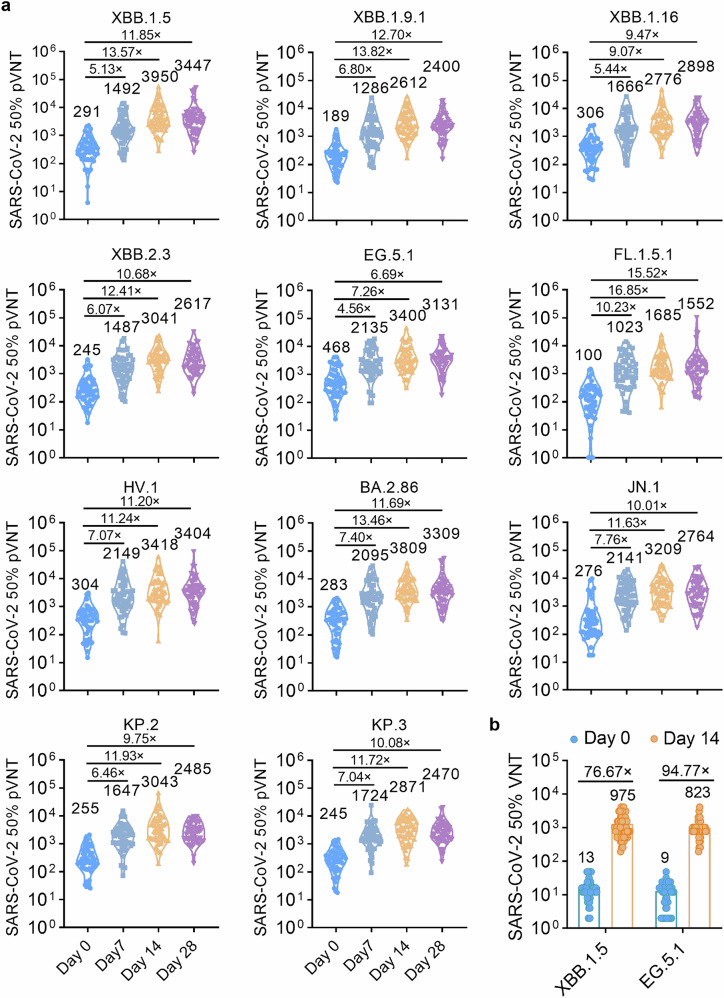
RBD_XBB.1.5_-HR vaccine improves neutralizing activities in human participants. **a** A total of 54 participants in age from 21 to 71 with (*n* = 15) or without underlying diseases (*n* = 39) were recruited into the clinical investigator-initiated trial (IIT) to evaluate the immunogenicity of RBD_XBB.1.5_-HR vaccine after sequential booster vaccination. The plasma samples were collected on days 7, 14 and 28 for pseudovirus neutralization assay (*n* = 54 samples). **b** The neutralizing antibodies in sera collected on day 14 against authentic XBB.1.5 and EG.5.1 viruses (*n* = 54 samples). Data are presented as geometric mean values with SD

The secondary objective is to evaluate the immunogenicity after sequential booster vaccination with the RBD_XBB.1.5_-HR vaccine in human participants in this trial. Sera samples were planned to be collected on 0-, 7-, 14- and 28-days post booster immunization for neutralization assays. Pseudovirus neutralization assays demonstrated that the RBD_XBB.1.5_-HR vaccine rapidly and significantly increased neutralizing antibodies against all XBB-lineage subvariants on day 7 after injection, further enhancing the neutralization responses on days 14 and 28 (Fig. 7a). Compared to pre-immunization, 50% neutralization GMTs on day 7 after vaccination against XBB.1.5, XBB.1.9.1, XBB.1.16, XBB.2.3, EG.5.1, FL.1.5.1 and HV.1 were improved by 5.13-, 6.80-, 5.44-, 6.07-, 4.56-, 10.23-, and 7.07-fold, respectively, and on day 14 were improved by 13.57-, 13.82-, 9.07-, 12.41-, 7.26-, 16.85-, and 11.24-fold, respectively. We next assessed whether the BA.2.86 and JN.1-derived variants could extensively escape the neutralizing effects of our monovalent RBD_XBB.1.5_-HR vaccine. Our results demonstrated that the vaccine induced robust neutralization against JN.1 and its descendants, KP.2 and KP.3 included. The GMTs of neutralizing antibodies against BA.2.86, JN.1, KP.2, and KP.3 on days 14 post-immunization were 3809, 3209, 3043, and 2871, respectively, representing increases of 13.46-, 11.63-, 11.93-, and 11.72-fold compared to sera samples collected pre-immunization. On day 28 post-immunization, the GMTs against these subvariants ranged from 2470 to 3309, further supporting the strong protective efficacy of our RBD_XBB.1.5_-HR vaccine in humans.

An authentic neutralization assay was also performed and found similar enhancements in neutralization against XBB.1.5 and EG.5.1 live viruses (Fig. 7b). These preliminary clinical results suggest that the recombinant RBD_XBB.1.5_-HR protein vaccine may serve as a safe and effective booster for humans for COVID-19 prevention, including the elderly and those individuals with underlying diseases.

## Discussion

Considering the near-complete global prevalence of Omicron JN.1- and XBB lineage-derived subvariants, characterized by their significant immune evasion following prior vaccination and breakthrough infection,^1,14–16,39–41^ alongside the phenomenon of imprinted immunity,^17,18^ there is an urgent requirement for the next-generation replacement of COVID-19 vaccine specifically tailored to target newly emerged subvariants. In alignment with the FDA’s recommendation on June 16, 2023, to use a monovalent XBB descendent lineage as the new vaccine antigen, several pharmaceutical companies have updated the formulations in vaccine to include antigens from the XBB.1.5 variant.^18,25,31^ In the current study, utilizing the self-assembly feature of HR,^32^ we successfully expressed a trimeric recombinant protein RBD_XBB.1.5_-HR. Our findings indicate that the adjuvanted RBD_XBB.1.5_-HR vaccine, administered either as a standalone vaccine or as an additional booster shot, is capable of eliciting a robust and sustained humoral and cellular immune response against JN.1, BA.2.86 and XBB-lineage subvariants. Furthermore, immunization with the RBD_XBB.1.5_-HR vaccine confers effective protective immunity against infection with the live EG.5.1 variant in the local upper and lower respiratory tract. Remarkably, the RBD_XBB.1.5_-HR vaccine exhibits favorable safety and tolerability profiles in human participants and demonstrates excellent ability to induce strong neutralizing potency against XBB-lineages, JN.1 and recent KP.2 and KP.3 subvariants, highlighting its potential for emergency use as a boost shot in clinical settings against the circulating Omicron variant. Currently, our RBD_XBB.1.5_-HR monovalent vaccine has received emergency use approval in China from the Chinese Center for Disease Control and Prevention.

Concerning presently available vaccines, including mRNA and inactivated vaccines, which have proved efficient in preventing symptomatic and severe infections, it is noteworthy that individuals with a history of vaccination and breakthrough infection exhibit substantial reductions in neutralizing activities, particularly attributable to the XBB and BA.2.86 family of SARS-CoV-2 lineages.^42,43^ In addition, the phenomenon of immune imprinting introduces the risk that vaccines containing components from previous variants may impede the redirection of neutralization responses toward new emerged subvariants.^12,17–20^ In face of such situation, the FDA advocates the development of future COVID-19 vaccines featuring monovalent XBB-lineage antigens. Therefore, we developed a trimeric recombinant protein vaccine derived from the XBB.1.5 RBD, exhibiting commendable immunogenicity in both animal models and human subjects, thereby indicating its clinical promise.

Moderna and Pfizer/BioNTech have updated the targets on the spike of XBB.1.5 subvariant to develop updated monovalent mRNA vaccine.^18^ This booster has shown strong efficacy in inducing high levels of neutralizing antibody titers against XBB and JN.1 subvariants.^22^ However, given the global use of mRNA vaccines, using a heterologous approach with a different protein-based platform for boosting may offer additional unique benefits over repeated homologous mRNA boosters. Our findings demonstrate that heterologous vaccination with the protein-based RBD_XBB.1.5_-HR vaccine, following three doses of either IV or mRNA vaccines, enhanced neutralizing activity in animal models. However, one limitation of our study is that we used BA.5-derived mRNA and WT-derived inactivated vaccines as homologous controls and did not perform a direct comparison of the efficacy between mRNA vaccines (e.g., Pfizer and Moderna) based on the XBB.1.5 spike sequence as homologous boosters and our RBD_XBB.1.5_-HR protein vaccine as a heterologous booster. Therefore, the results from the heterologous vaccination experiments suggest that the RBD_XBB.1.5_-HR protein vaccine may serve as a promising candidate for booster shots. Additionally, considering the distinctions in vaccine doses and the heterogeneous hybrid immune backgrounds within the human population, the definite effectiveness of the RBD_XBB.1.5_-HR monovalent vaccine as a functional booster needs further investigation in larger-scale clinical trials.

In recent years, substantial efforts have been devoted to the development of next generation of vaccines against COVID-19 in preclinical studies, however, the shift of vaccines towards clinical application for safeguarding humanity still holds paramount importance. It is worth noting that our monovalent RBD_XBB.1.5_-HR vaccine, characterized by its favorable tolerability and robust immunogenicity, demonstrates a capacity to enhance sera neutralizing capabilities in all human participants (100% positive) who have previously received COVID-19 vaccine. Older adults, especially those with stable comorbidities, face susceptibility to both SARS-CoV-2 infections and their related complications.^22^ Furthermore, the elderly population tends to exhibit diminished responses to vaccination, marked by reduced neutralizing,^44–47^ potentially stemming from immunosenescence, characterized by a compromised adaptive immune response, chronic elevation of inflammatory cytokine levels, and dysregulated cytokine production.^48,49^ Given the alterations in the aging immune system, assessments of vaccine effectiveness in the elderly population should be carried out separately.^48^ Therefore, our clinical Investigator-Initiated Trial (IIT) involved the recruitment of 19 (35.2%) older adults aged 60 and above and found RBD_XBB.1.5_-HR vaccine boost provides effective protective immunity in elderly participants and those with underlying diseases (*n* = 15, 27.8%), underscoring the promising clinical application value of the monovalent RBD_XBB.1.5_-HR vaccine.

Given that the sample size consisted of merely 54 participants, the conclusions derived from these data are restricted. Therefore, the findings should be regarded as preliminary in terms of the safety and immunogenicity of the RBD_XBB.1.5_-HR monovalent vaccine. The small number of recruited participants also barred us from undertaking stratified analyses of safety and immunogenicity on the basis of factors such as age, gender, or underlying health conditions. While we have shown that the RBD_XBB.1.5_-HR vaccine induces long-lasting protective immune responses in mice, lasting up to 40 weeks post-vaccination, further monitoring of neutralizing activity in human subjects is essential to evaluate the vaccine’s effectiveness against circulating variants in time to come. It is of importance to note that the RBD_XBB.1.5_-HR vaccine’s safety and immunogenicity are currently investigated in a Phase 2 clinical trial, with a Phase 3 trial already underway (data not yet published). The vaccine has also received emergency use authorization in China. Despite these limitations, the present study emphasizes that the RBD_XBB.1.5_-HR vaccine is a safe and promising subunit protein-based booster, capable of providing immunity in individuals with a hybrid immune profile.

## Materials and methods

### Ethics statement

All mouse and rat vaccination experiments were approved by the Institutional Animal Care and Use Committee of Sichuan University (Approved number: 20230207001).

Animal procedures involving live SARS-CoV-2 EG.5.1 virus challenges were reviewed and approved by the Institutional Animal Care and Use Committee of the Institute of Medical Biology, Chinese Academy of Medical Sciences, and conducted in the ABSL-4 facility at Kunming National High-level Biosafety Primate Research Center (Approved number: DWLL202409015).

Human participant procedures in the investigator-initiated trial (IIT) were reviewed and approved by the Ethics Committee of West China Hospital (Registration number: ChiCTR2300068248). All participants provided written informed consent.

### Preparation of recombinant trimeric RBD_XBB.1.5_-HR protein

The recombinant trimeric protein was constructed by exploiting the self-assembling properties of the HR sequence, as described in our previous study.^32^ We linked the RBD sequence (320–545 aa) from the XBB.1.5 variant with HR1 (916–966 aa) and HR2 (1157–1203 aa) from the S2 subunit in tandem. To prevent undesired dimerization, we introduced a C538S mutation in the RBD to eliminate inter-chain disulfide bonds. The Bac-to-Bac Baculovirus Expression System was used to express the recombinant RBD_XBB.1.5_-HR protein.

The protein sequence was engineered with a GP67 signal peptide for protein secretion, a Trx tag that assists in folding, a 6×His tag that facilitates purification, and an enterokinase (EK) cleavage site that enables tag removal. The gene was cloned into the pFastBac1 vector, which was then used to generate recombinant bacmids in *E. coli DH10b* cells. Subsequently, the bacmids were transferred to Sf9 cells for protein expression.

After expression, the protein was cleaved by EK protease and purified using a Superdex 200 Increase 10/300 GL column. SDS-PAGE, Coomassie blue staining, as well as western blotting, were used to verify the protein purity. Finally, the purified recombinant RBD_XBB.1.5_-HR proteins were mixed with MF59-like oil-in-water adjuvant (composing of squalene, Tween 80 and Span 85) at a volume ratio of 1:1 to formulate the vaccines.

### Vaccination of mice and rats

Specific pathogen-free (SPF) female National Institute of Health-Swiss (NIH) mice and SD rats (6–8 weeks old) were obtained from Beijing Vital River Laboratory Animal Technologies Co., Ltd. (China) and housed in SPF conditions (21–25 °C; 30–70% of humidity; 12 h light/dark cycle) at the State Key Laboratory of Biotherapy, Sichuan University. Mice (*n* = 6/group) were randomly assigned to receive intramuscular injections of PBS, adjuvant, or 10 μg of monovalent RBD_XBB.1.5_-HR vaccine, as indicated in each figure legend. SD rats (*n* = 6/group) were immunized with 30 μg (low-dose group) or 60 μg (high-dose group) of RBD_XBB.1.5_-HR vaccine, respectively. Immunizations were performed on weeks 0, 3, and 6, with serum samples collected 8 or 43 weeks after the first dose.

For the assay of heterologous-booster immunization, the mice were intramuscularly immunized three times with 50U of inactivated virus vaccine (IV, VacKMS1), manufactured by Medical Biology (IMB) in Kunming,^50^ or a BA.5 variant-derived full-length spike mRNA vaccine^51^ with the several proline mutations^52^ at 0, 2 and 8 weeks. Among them, one group were received one dose of monovalent RBD_XBB.1.5_-HR vaccine at 16 weeks (IV×3 > RBD_XBB.1.5_-HR Vac, or mRNA×3 > RBD_XBB.1.5_-HR Vac). At the same time, the mice were immunized with four doses of IV (IV×3 > IV) or four doses of mRNA vaccine (mRNA×3>mRNA) were set as control groups. Sera samples were planned to be collected on day 14 after the fourth dose.

### Binding antibodies assay by enzyme-linked immunosorbent assay (ELISA)

To measure antigen-specific IgG antibodies, 1 μg/mL RBD_XBB.1.5_-HR proteins in carbonate coating buffer was applied to coat the 96-well plates (NUNC-MaxiSorp, Thermo Fisher Scientific) overnight at 4 °C. After three washes with PBST (PBS with 0.1% Tween-20), the plates were blocked with 1% BSA containing PBST for 1 hour at 37 °C. Two-fold diluted sera samples (100 μL/well) were then added in to the wells and incubated at 37 °C for 1 h. After 5 washes with PBST, HRP-conjugated anti-mouse IgG antibodies (1:10,000 dilution, Southern Biotech, Cat: 0107-05), were added and kept at 37 °C for 1 h. 100 μL of 3,3′,5,5′-tetramethylbenzidine (TMB) substrate was added following 5 washes. The reaction was stopped in 10 minutes with 1 M H_2_SO_4_, and absorbance of each well was measured using a microplate reader (Spectramax ABS, Molecular Devices) at 450 nm.

### SARS-CoV-2 pseudovirus neutralization assay

Pseudovirus neutralization of serum samples from mice, rats and human participants was performed as described.^32^ A variety of SARS-CoV-2 pseudoviruses, including Delta, BA.2.75, BA.5, BF.7, BQ.1, BQ.1.1, XBB, XBB.1.5, XBB.1.9.1, XBB.1.16, XBB.1.16.6, XBB.2.3, EG.5.1, FL.1.51.1, HV.1, BA.2.86, JN.1, KP.2, and KP.3, were provided by Genomeditech. Inactivated sera samples were three-fold diluted and incubated with each pseudovirus in plates (WHB-96-03, WH Biotech) at 37 °C for 1 h. Human ACE2 expressing HEK-293T cells (293 T/ACE2) were added to the wells to a final concentration of 1.5 × 10^4^ cells/well and incubated at 37 °C for 2 days to allow luciferase expression in cells. After discarding the supernatant, 100 μL of lysis reagent containing luciferase substrate (Beyotime, RG005) was added to each well. Within 10 min, Luminescence intensity was measured using a PerkinElmer multi-mode microplate reader (PerkinElmer, USA). Calculation was conducted using GraphPad Prism 8.0.2 to analysis 50% neutralization titers (EC50).

### Live SARS-CoV-2 virus neutralization assay

Authentic virus neutralization was conducted to evaluate the neutralizing antibody titers in sera samples against live Delta, BA.5.2.48, XBB.1.5, XBB.1.16, EG.5.1, and JN.1 SARS-CoV-2 variants. The sera were diluted and mixed with the respective live virus, then added to Vero E6 cell plates (5 × 10^4^ cells/well) and incubated for 2–3 days at 37 °C. Cytopathogenic effects (CPE) were visualized under a microscope. The neutralizing antibody titers were determined by calculating the EC50 (the dilution that inhibits 50% of the viral activity).

### Enzyme linked immunospot assay (ELISpot)

To measure antibody-secreting cells (ASCs), sterile 96-well ELISpot filter plates were coated overnight at 4 °C with 3 μg/mL of XBB.1.5 or JN.1 RBD protein, then culture medium was added to each well to block for 2 h in a 37 °C incubator. Cells from bone marrow and spleen tissues were added into the wells and incubated overnight at 37 °C with 5% carbon dioxide. After washing the plates with PBS, HRP-conjugated goat anti-mouse IgG (1:10,000) was applied for 2 h at room temperature. Following with 5 additional washes, 100 μL TMB ELISpot substrate was added to form spots, which were captured and counted using an AID ELISpot Reader.

For detecting IFN-γ-secreting lymphocytes, IFN-γ ELISpot plates were firstly washed four times with PBS. After adding 100 μL of complete 1640 medium per well and a 1 h incubation at room temperature, splenic lymphocytes (2 × 10^5^ cells/well) were added and stimulated overnight with peptide pools from Omicron spike proteins (XBB.1.5, JN.1). The plates were washed after removing the cells and incubated for 2 h with biotinylated detection antibody (7-B6-1, 1 μg/mL). The wells were then incubated with Streptavidin-ALP (1:1000) for 1 h at room temperature. After washing, BCIP/NBT-plus substrate solution was applied to each well to form spots. Once spots appeared, development was halted with water rinsing. The spot in the plates were scanned and counted using an AID ELISpot Reader.

### Flow cytometry

Lymphocytes from spleens from the vaccinated mice were isolated, seeded at a final concentration of 1 × 10^6^ cells/well in 12-well plates, and cultured with RPMI-1640 medium (containing 10% FBS, 100 μg/ml streptomycin, 100 U/ml penicillin, 50 μM β-mercaptoethanol, 1 mM pyruvate, as well as 20 U/ml mouse IL-2), followed by stimulating with 1 μg/ml peptide pools from full-length XBB.1.5 or JN.1 spike protein for 12 h. Cells were pretreated with brefeldin A for 6–8 h, then stained with anti-CD3 (PerCP/Cyanine5.5), anti-CD8a (APC), anti-CD4 (FITC), and anti-CD44 (Brilliant Violet 510) for 30 min at 4 °C in dark. After 2 washes, the lymphocytes were fixed and permeabilized (Fixation/Permeabilization Kit, BD Biosciences) and stained with anti-IFN-γ (PE) and anti-TNF-α (Brilliant Violet 421) for 1 h at room temperature kept from light. Samples were analyzed by a Flow Cytometer (ACEA Biosciences) using NovoExpress software (version1.4.1).

For germinal center (GC) B cells, cells were stained with anti-CD3 (PerCP/Cyanine5.5), anti-CD19 (Brilliant Violet 605), anti-CD95 (PE/Cyanine7), and anti-GL7 (Pacific Blue). For Tfh cells, cells were stained with anti-CD19 (Brilliant Violet 605), anti-CD3 (PerCP/Cyanine5.5-), anti-CD4 (FITC), anti-CXCR5 (Brilliant Violet 421), and anti-PD-1 (PE). For LLPCs, cells were stained with anti-CD44 (FITC), anti-IgD (PE), anti-CD138 (Brilliant Violet 605), and anti-CD19 (Brilliant Violet 605). MBCs were detected with anti-CD3 (PerCP/Cyanine5.5), anti-CD19 (Brilliant Violet 605), anti-CD38 (PE/Cyanine7), and anti-GL7 (Pacific Blue). Frequencies of Tfh, LLPCs, GC B cells, and MBCs were analyzed on a NovoCyte Advanteon Flow Cytometer using NovoExpress 1.5.6.

Gating strategies for antigen-specific T memory cells, Tfh, GC B, MBCs and LLPCs were seen in the Supplementary information.

### Challenge of mice with live Omicron EG.5.1 viruses

NIH mice (6-8 weeks old) were divided into two groups and intramuscularly injected with 10 μg of monovalent RBD_XBB.1.5_-HR vaccine or adjuvant alone on days 0, 21, and 42. On day 63, all the mice were intranasally challenged with 1 × 10^6^ PFU of live EG.5.1 viruses. Daily monitoring was performed for bodyweight change and viral loads in throat swabs. All of the mice were euthanized on the fifth day following infection, and tissues were collected to assess pathological changes and viral loads. Genomic RNA (gRNA) in nasal turbinate, trachea, and lung tissues was quantified using reverse-transcription quantitative PCR (RT–qPCR), while hematoxylin and eosin staining (H&E) was applied to evaluated pathological changes in lung tissues. The sequences of primer and probe used for gRNA detection were as follows: 5’-GACCCCAAAATCAGCGAAAT-3’ (forward), 5’-TCTGGTTACTGCCAGTTGAATCTG-3’ (reverse), 5’-FAM-ACNGCCGCATTACGTTTGGTGGACC-BHQ1-3’ (probe).

All animal procedures were reviewed and approved by the Institutional Animal Care and Use Committee of the Institute of Medical Biology, Chinese Academy of Medical Sciences, and were conducted in the ABSL-4 facility of the Kunming National High-level Biosafety Primate Research Center.

### Study design of evaluation of safety and immunogenicity of RBD_XBB.1.5_-HR vaccine in human participants

An investigator-initiated trial (IIT) was performed in order to evaluate the safety and immunogenicity of the monovalent RBD_XBB.1.5_-HR vaccine in human participants, including those with underlying conditions (Registration number: ChiCTR2300068248). The study was approved by the Biomedical Ethics Review Committee of West China Hospital, Sichuan University, and all participants provided written informed consent. Participants were required to test negative for SARS-CoV-2 antigen to avoid interference from prior infections. Those with a positive antigen test were excluded.

In total, 54 participants aged 18 and older with a history of COVID-19 vaccination (and >3 months since the last dose) were enrolled. Serum samples were scheduled to collect before vaccination (pre-immunization control) and participants received one dose of the RBD_XBB.1.5_-HR vaccine. They were asked to complete a diary to report adverse reactions online within 7 days post-vaccination. Blood samples were scheduled to be collected on days 7, 14, and 28 for neutralizing antibody assays, and plasma was stored at −80 °C.

### Statistical analyses

Statistical analyses were conducted using Prism 8.0.2 (GraphPad). Data are presented as geometric mean ± SD or mean ± SEM, as specified in the figure legends. Significance was determined using one-way or two-way ANOVA, as indicated in the figure legends. Comparisons between two groups were made using unpaired Student’s *t*-tests. *P* values < 0.05 were considered significant. ****P < 0.0001; ***P < 0.001; **P < 0.01; *P < 0.05; ns, not significant.

## Supplementary information

Supplementary Materials

## Data Availability

All data that support the findings of this study are available with the main text and Supplementary information.
