# Supplementary material for: A recombinant protein vaccine induces protective immunity against SARS-CoV-2 JN.1 and XBB-lineage subvariants
Source: Signal Transduct Target Ther. 2025 Feb 26;10:58. doi: 10.1038/s41392-025-02154-6 (PMC11862015; doi:10.1038/s41392-025-02154-6)
Supplement: Supplementary file 1 — Supplementary Materials [file 41392_2025_2154_MOESM1_ESM.docx]

Supplementary Materials for

A recombinant protein vaccine induces protective immunity against SARS-CoV-2 JN.1 and XBB-lineage subvariants

Jingyun Yang ^1^†, Weiqi Hong^1^†, Huashan Shi^1^†, Zhenling Wang^1^†,Cai He^1^†, Hong Lei^1^†, Hong Yan^1^†, Aqu Alu^1^, Danyi Ao^1^, Zimin Chen^1^, Yanan Zhou^2^, Hao Yang^2^, Yun Yang^2^, Wenhai Yu^2^, Cong Tang^2^, Junbin Wang^2^, Bai Li^2^, Qing Huang^2^, Hongbo Hu^1^, Wei Cheng^1^, Haohao Dong^1^, Jian Lei^1^, Lu Chen^1^, Xikun Zhou^1^, Li Yang^1^, Wei Wang^1^, Guobo Shen^1^, Jinliang Yang^1^, Zhiwei Zhao^1^, Xiangrong Song^1^, Qiangming Sun^2^, Youchun Wang^2^, Shuaiyao Lu^2^*, Jiong Li^1^*, Guangwen Lu^1^*, Weimin Li^3^*, Yuquan Wei^1^*, Xiawei Wei^1^*

Correspondence to: xiaweiwei@scu.edu.cn

**This PDF file includes:**

Figures. S1 to S4

Figure. S1.

Supplemental Figure 1. Gating strategy for antigen-specific T memory cells, T follicular helper (Tfh), germinal center B (GC B), memory B cells (MBCs) and long-lived plasma cells (LLPCs).

Figure. S2.


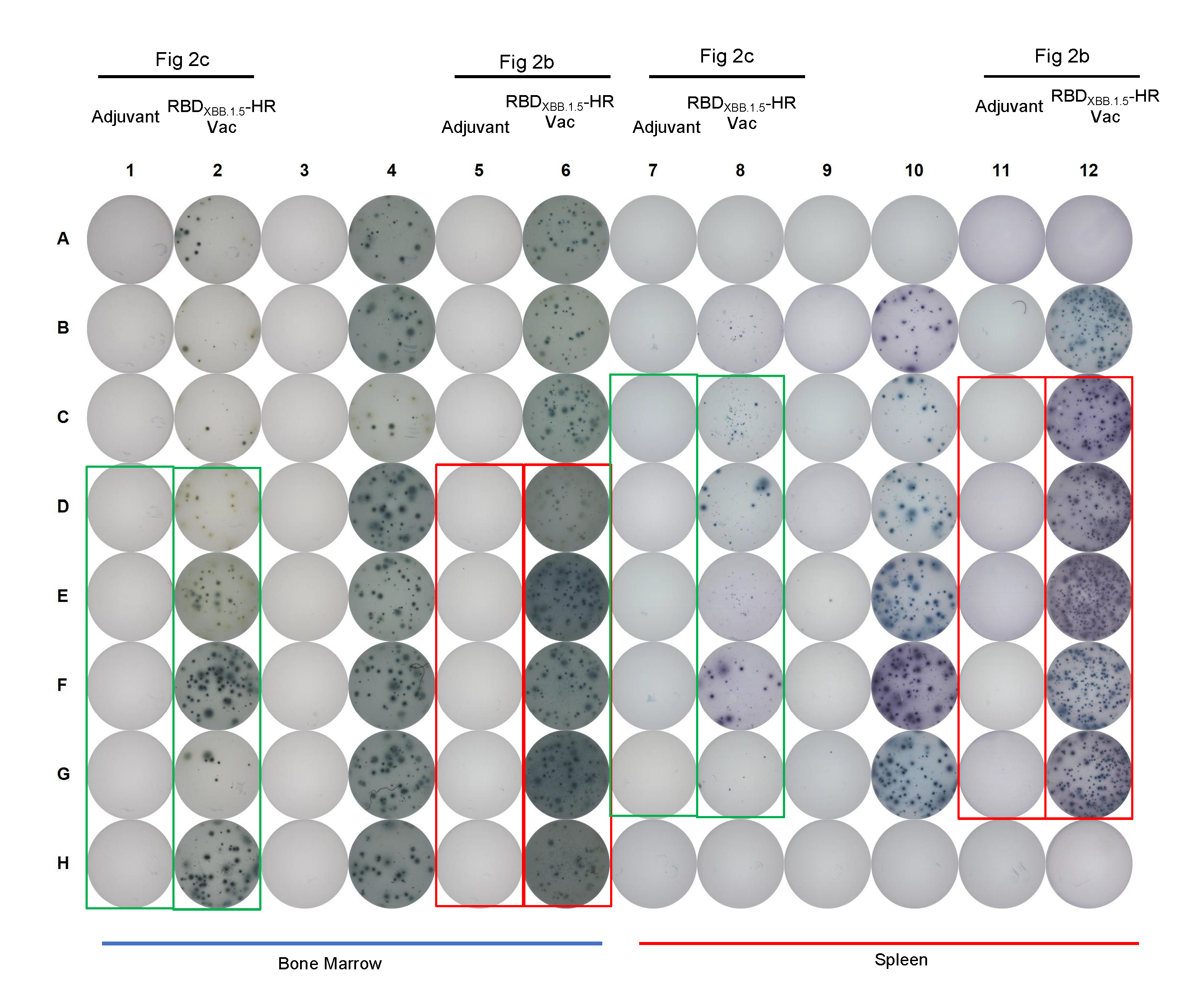


Supplemental Figure 2. Original image of ELISPOT plate in figure 2.

Figure. S3.


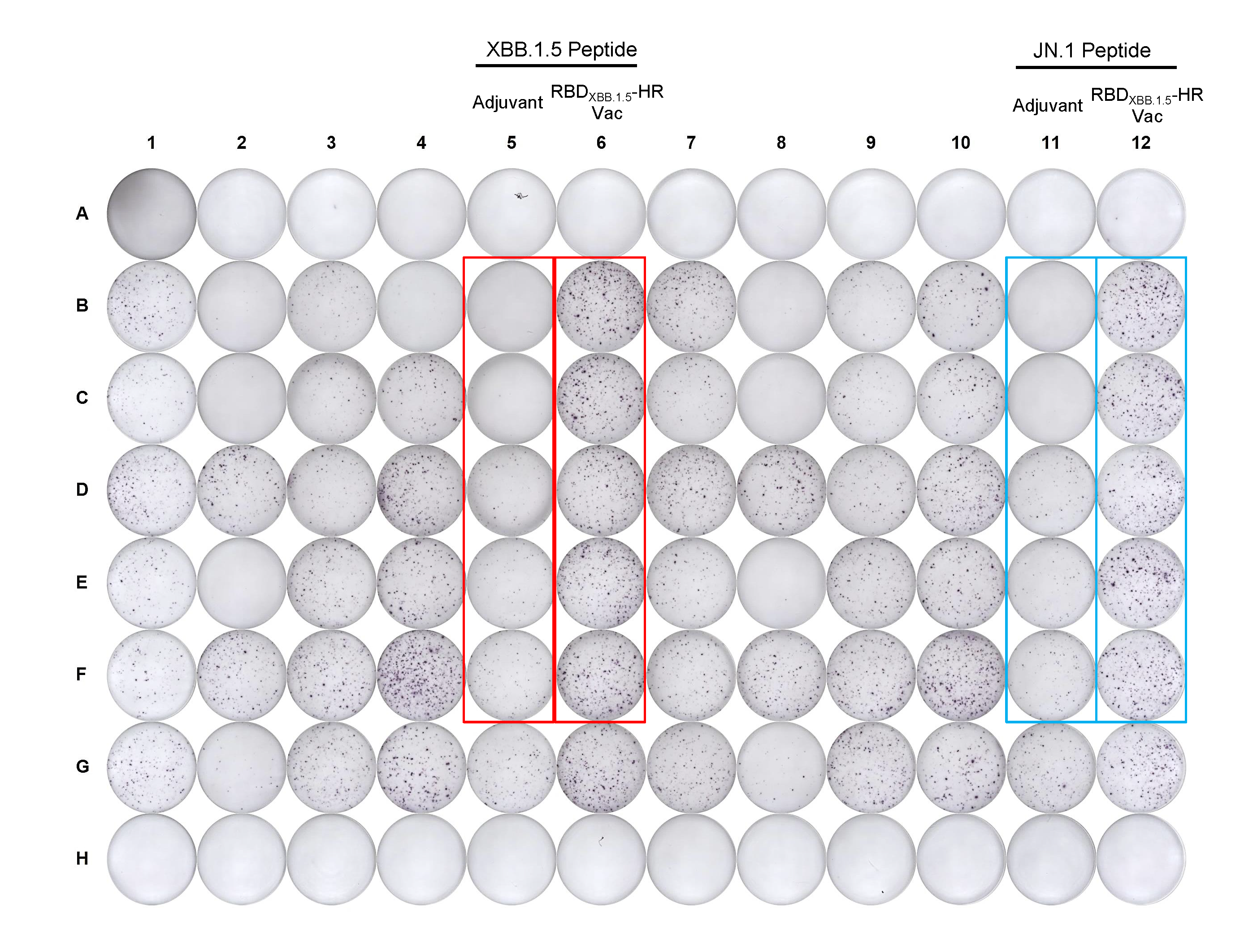


Supplemental Figure 3. Original image of ELISPOT plate in figure 3.


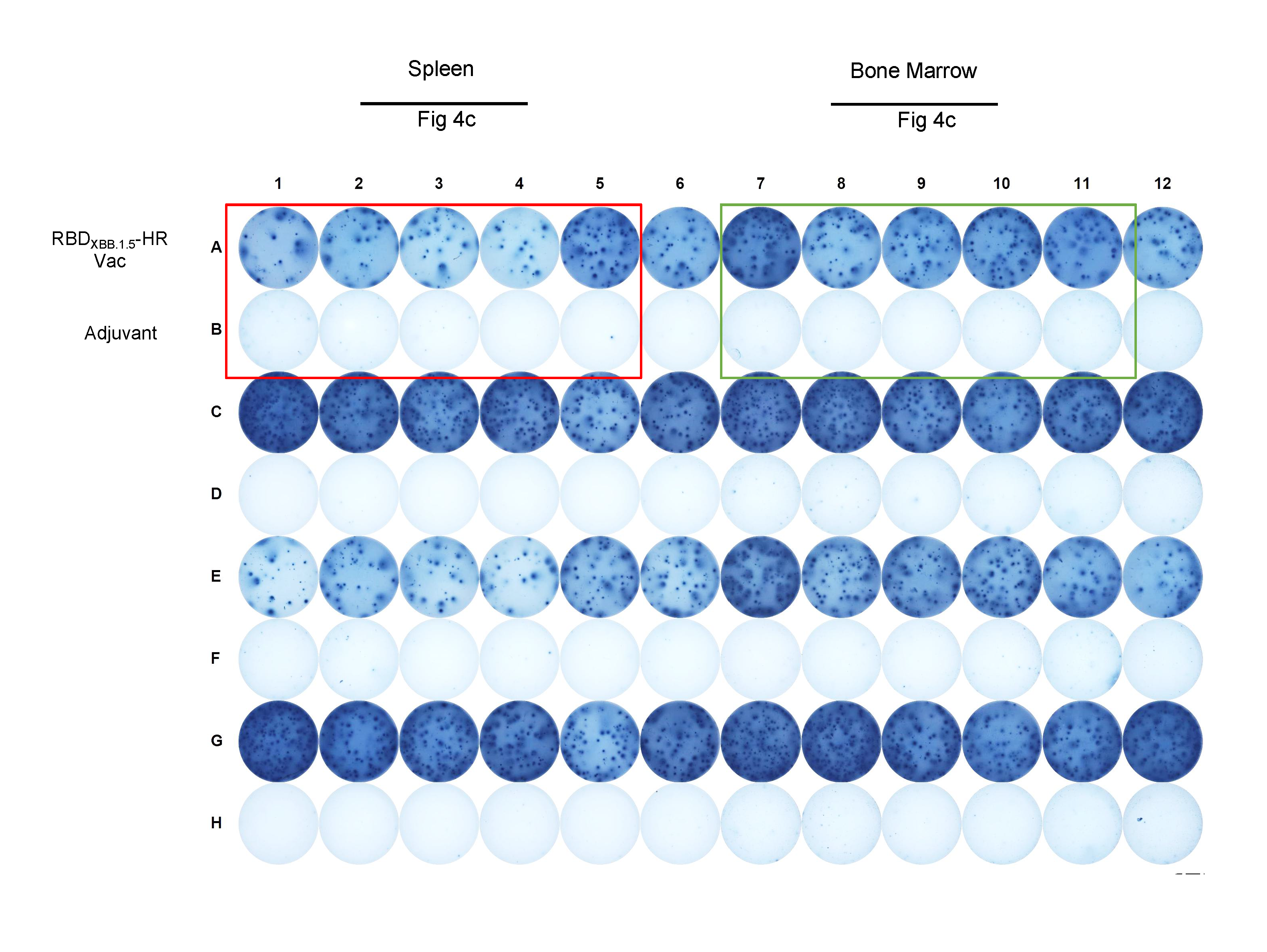
Figure. S4.

Supplemental Figure 4. Original image of ELISPOT plate in figure 4.
